# Risk factors for, and prediction of, exertional heat illness in Thoroughbred racehorses at British racecourses

**DOI:** 10.1038/s41598-023-27892-x

**Published:** 2023-03-14

**Authors:** Leah E Trigg, Sally Lyons, Siobhan Mullan

**Affiliations:** 1 Bristol Veterinary School, University of Bristol, Langford House, Langford, Bristol BS40 5DU UK; 2 British Horseracing Authority, 75 High Holborn, London, WC1V 6LS UK; 3 School of Veterinary Medicine, University College Dublin, Belfield, Dublin 4, Ireland

**Keywords:** Animal physiology, Predictive medicine, Risk factors, Environmental health

## Abstract

The development of exertional heat illness (EHI) is a health, welfare and performance concern for racehorses. However, there has been limited multivariable assessment of the possible risk factors for EHI in racehorses, despite such information being vital for regulators to effectively manage the condition. Consequently, this study aimed to identify the risk factors associated with the occurrence of EHI in Thoroughbred racehorses and assess the ability of the risk factor model to predict the occurrence of EHI in racehorses to assist in early identification. Runners at British racecourses recorded in the British Horseracing Authority database between 1st July 2010 and 30th April 2018 were used to model the probability that a horse would present with EHI as a function of a suite of environmental, horse level and race level factors. EHI was reported in 0.1% of runners. Race distance, wet bulb globe temperature, preceding 5-day temperature average, occurrence of a previous EHI incident, going, year and race off time were identified as risk factors for EHI. The model performed better than chance in classifying incidents with a mean area under the receiver operating characteristic curve score of 0.884 (SD = 0.02) but had a large number of false positives. The results provide vital evidence for industry on the need to provide appropriate cool down facilities, identify horses that have repeated EHI incidents for early intervention, and collect new data streams such as on course wet bulb globe temperature measurements. The results are especially relevant as the sport is operating in a changing climate and must mitigate against more extreme and longer spells of hot weather.

## Introduction

Exercising racehorses confront a thermoregulatory challenge. To maintain an appropriate internal temperature, they must balance heat input from the surrounding environment and heat generation from the internal conversion of stored energy into movement, with the activation of heat loss mechanisms such as sweating^1–3^. It is estimated that heat production in a Thoroughbred racehorse will be as much as 1250 kJ min$^{-1}$^1,4^. Experimental evidence suggests such heat production can increase the temperature of blood in the cartoid artery by 2.3 $^{\circ }$C after just 3 min^4^. In the event that exercising racehorses cannot maintain an internal heat balance by losing such heat to the environment, internal temperature will continue to rise and horses are at risk of exertional heat illness (EHI)^1,3,5^. Particularly, internal temperatures of 40 °C are thought to be the critical thermal maximum above which there is a high likelihood of protein denaturation and cell death, as well as a cascade of physiological harm^3,6,7^. Exertional heat illness falls on a continuum, with symptoms ranging from a minor elevation in a horse’s respiratory rate and heart rate, to severe central nervous system dysfunction or death^2,3^. In ponies, a redistribution of blood flow from working muscles to thermo-regulatory tissues, is thought to contribute to the observed early onset of fatigue in mildly hot compared to thermoneutral conditions^2,8^. Treatment of EHI is primarily through early identification and rapid agressive cooling of horses with cold water, as well as drugs to calm erratic behaviour and ensure the safety of personnel treating horses with EHI^3^. Given such negative impacts, EHI is a health, welfare and performance concern for racehorses. Horse racing regulators worldwide aim to protect racehorses from such deleterious impacts^9,10^. However, such regulatory bodies need to understand the risk factors for EHI to best mitigate harm.

There are a number of factors that could influence the thermal balance of an exercising horse. Speed and length of exercise^11^, the level of hydration^12^, level of acclimatisation^13–15^, fitness level of horse^16^, age^17^, sex^18^ and breed^19^ have been implicated as factors in the occurrence of heat related illness in horses. Evidence suggests that older horses are more at risk of heat related illness due to impairment of the cardiovascular system^2,17,18^. Geldings and mares were also at higher risk than uncastrated males in an assessment of Japanese Thoroughbreds running over the flat^18^. However, of key consideration is the environmental conditions under which horses are exercising^20^. When comparing horses undertaking the cross country phase of a 3-day event in a cold or hot climate, the rectal temperatures, heart and respiratory rates of horses were all significantly higher under hot environmental conditions^21^. High temperatures act as a direct heat input but they also influence the efficiency of heat loss mechanisms. Conduction, convection and radiative heat loss are driven by a temperature gradient, whereby the environment is cooler than the skin or respiratory heat loss surface^1,2^. In contrast, evaporative cooling through sweating is the primary heat loss mechanism when this temperature gradient is too weak for other mechanisms to be effective^1–3^. However, humid conditions can prevent the evaporation of sweat. Under hot and humid conditions, Thoroughbred racehorses exercising on a treadmill at 50% $VO_{2max}$ to a pulmonary artery temperature of $41.5^{\circ }$C can exercise for significantly shorter time than horses in hot and dry conditions^22^. The rate of blood temperature increase, muscle temperature and heart rate were all significantly higher for horses exercising under hot and humid conditions^22^.

In the United Kingdom (UK) and globally, the occurrence of such temperature extremes is becoming more likely. The top 10 warmest years in the UK since 1884 have all occurred since 2002 and four national UK high temperature records were set in 2019 including a new all time high of 38.7 °C^23^. Such changes are also evident in Japan, where the prevalence of EHI per year has been increasing; the prevalence between 2015 and 2018 (0.07%) was significantly higher than between 1999 and 2014 (0.04%)^24^. At a global scale, every decade in the last 40 years has been warmer than the decade before and global surface temperature has increased faster since 1970 than any other 50 year period in the last 2000 years^25^. Each of the Intergovernmental Panel on Climate Change future climate scenarios predict an increase in global surface temperature in the future^25^. However, the impact of climate change on professional sport has not received much attention^26^. Horse racing particularly, as an outdoor sport reliant on ground and weather conditions, is vulnerable to future climate extremes. As a result, regulators and racecourses will increasingly have to make decisions and deploy resources in response to warm weather extremes to reduce the risk of EHI in racehorses. However, they lack the evidence on which to base EHI policies.

There are few multivariable studies of the possible risk factors for EHI in racehorses. Takahashi et al.^18^ examined the risk factors for EHI in Thoroughbred racehorses running over the flat in Japan. They reported that the wet bulb globe temperature index, sex, race distance, age and body-weight were associated with greater odds of EHI. In Australia, race distance, humidity, wind speed and temperature were related to the percentage of EHI incidents^20^. However, there has been no assessment of risk factors for EHI in different climatic regions or under different racing regimes such as those races run over obstacles. Therefore, this study aims to identify the risk factors associated with the occurrence of EHI in Thoroughbred racehorses taking part in jump and flat races at racecourses in Britain, a temperate climatic region. Given the evidence of a changing climate, there is a vital need to assess the risk factors and develop tools to aid decision makers in addressing EHI in racing jurisdictions around the world. Furthermore, early detection of EHI in afflicted horses followed by aggressive cooling using cold water is vital for a good outcome^1,3^. Consequently, this study also aims to go further than previous studies and assess the ability of the risk factor model to predict the occurrence of EHI in Thoroughbred racehorses to assist in early identification.

## Methods

We examined all runners at British racecourses recorded in the British Horseracing Authority (BHA) database between 1st July 2010 and 30th April 2018. There were 704,434 runners during this time of which 702 resulted in an exertional heat illness (EHI) incident. This data was used to model the probability that horses would present with EHI to racecourse veterinary officers as a function of a suite of possible risk factors (Table 1). The horses were not directly involved in the study. We utilised data collected by the BHA in its role as regulator, and the BHA gave permission for the use of the data as part of this research. The data were collected in accordance with the guidelines and ethical policies of the BHA.

### Exertional heat illness incidents

Incidents of exertional heat illness were extracted from the database based on the classification of incidents recorded by BHA racecourse veterinary officers in the regulatory recording system. Cases were defined either as ‘heat stress’ or ‘exhaustion’ with heat listed as a contributory factor. This study uses the term exertional heat illness (EHI) to refer to all exercise induced heat related problems presented to, or identified by, veterinary officers and recorded in the database.

**Table 1:** Proposed explanatory factors included in the maximal model prior to backward stepwise variable selection by 5-fold cross validation.

Race factors	Horse factors	Environmental factors
Race distance	Age	Wet bulb globe temperature
Race type	Sex	Previous 5-day temperature average
Race-off time	Previous incident	
Year		
Going		

### Horse level risk factors

There were three horse level risk factors included in the model; age, sex and whether the horse had a previous EHI incident. In the BHA database horses are classified as either filly, colt, gelding, stallion or rig. There were only 7 rig horses, and therefore, these were removed from the dataset. There are anecdotal observations that horses often have repeated incidents of EHI^2^. As a result, whether the horse had one or more EHI incidents was included as a binary variable. This variable might also help to encompass some of those horse level factors for which there was no data.

### Race level risk factors

There is detailed information about each race run held within the BHA regulatory database. The race distance in yards, race type (jump or flat), race off time (pre or post 5 pm), year and going (firm, good, heavy, soft and standard) were included in the model. In order to assist in model convergence, going was reduced from 10 to 5 categories (Supplementary Table S1). Going was grouped as either firm, good, soft or heavy for turf tracks and standard for all weather surfaces. There were only three all weather race meets run over fast ground. Therefore, these were removed from the dataset. Given that the type of going also suggests the type of surface used, the race surface was not included as a separate variable in the model. The race off time splits races run during the day (Pre 5 p.m.) and in the evening (Post 5 p.m.). Evening races potentially have cooler and more favourable environmental conditions. Other racing jurisdictions have reported rising EHI incidents over time^24^. Therefore, year of race was included in the model to assess any changes in EHI incidents over time.

### Environmental risk factors

The environmental risk factors assessed in the model were wet bulb globe temperature (WBGT) and the average temperature at the racecourse in the five days before a race. WBGT is an index of environmental load originally developed for assessing suitable conditions for human military recruits^27^. It is a composite value of humidity, solar radiation, temperature and wind speeds, all of which are thought to influence the environmental stress imposed on an exercising equine^20,28^. In this context, it provides a useful combination of otherwise co-linear environmental variables and has been shown to have a closer relationship to the environmental heat load than the addition of humidity and temperature values^29^. WBGT was not recorded at racecourses for the duration of this study. Therefore, the value was approximated from the relative humidity and dry globe temperature values using published equations by Stull^30^ and Schroter and Marlin^31^. Equation 1 calculates an approximation of wet bulb temperature ($T_{wb}$) from relative humidity (*H*) and air temperature (*T*)^30^. Equation 2 estimates the black globe temperature ($T_{g}$) from air temperature (*T*), and Eq. 3 calculates the WBGT index from the estimated wet bulb temperature ($T_{wb}$) and black globe temperature ($T_{g}$)^31^.

1 $$\begin{aligned} T_{wb}= & {} T atan[0.151977(H + 8.313659)^{0.5}] + atan(T + H) - atan(H - 1.676331) \nonumber \\{} & {} + 0.00391838(H)^{3/2} atan(0.023101H) - 4.686035 \end{aligned}$$

2 $$\begin{aligned} T_{g}= & {} 1.54T + 8.65 \end{aligned}$$

3 $$\begin{aligned} WBGT= & {} 0.7 T_{wb} + 0.3 T_{g} \end{aligned}$$

The estimation of WBGT, as described above, requires data on air temperature and relative humidity as input. Daily maximum air temperature and relative humidity values were used to calculate WBGT at the racecourse for the day of each race meet. The daily maximum air temperature was interpolated (nearest) from 5 km gridded surface climate observations of the UK generated by the Met Office^32^. The location of each racecourse was given by latitude and longitude. The 5 km grid cells provide an accurate assessment of weather at the racecourse. Relative humidity data was extracted from Met Office weather station data based on the postcode of the racecourse. To assess the influence of acclimatisation on the probability of exertional heat illness, the average maximum daily temperature of the preceding five days at the racecourse was included as an explanatory variable. Interactions between environmental variables were computationally intensive and did not yield significant results. Therefore, they were not explored further (Supplementary Methods).

### Statistical analysis

We modelled the binary response EHI/No EHI as a function of the above described risk factors using a binomial generalised additive mixed model (GAMM)^33^. This model allows for a non-linear relationship between the the response and predictors variables. The mean response is dependent on the explanatory variable through the sum of a number of smoothing functions and fixed effects. Random effects are used to model correlation between observations. While typically numerically more difficult to solve than a generalised linear mixed model (GLMM), diagnostic plots of the logit and continuous predictors suggested there may be some non-linearity and the GAMM converged more efficiently than a GLMM (Supplementary Fig. S1). The final model was selected by first fitting a maximal model with all variables described above. The minimum adequate model was selected using backward stepwise removal of variables with k-fold cross validation where k = 5^34,35^. The 5-fold cross validation method randomly assigns data to five datasets or folds. Each fold is used once for testing the ability of the model which was trained on the other four folds^35^. This ensures all data points are used to test the performance of the model^35^. Models were assessed using the Brier Score (Eq. 4) where $f_{t}$ is the forecast probability and $o_{t}$ is the outcome probability for the $t$th observation^36^. The Brier Score is a proper scoring rule and provides an assessment of the accuracy, equivalent to the mean square error, of the predicted probabilities^36^. The maximum score depends on the incidence of the event in the model. Therefore, the score is scaled by the maximum value to allow comparison of folds where $mean(p)$ is the average probability of the outcome (Eqs. 5, 6) with lower Brier values being preferred^35,36^. Continuous explanatory variables (race distance, WBGT, previous 5-day temperature average and age) were modelled as penalised cubic regression splines with shrinkage^37,38^. To assess the number of knots required, models were fitted with $k-1$, 10, 40 and 80 knots but there was only marginal variation between the results. GAMMs were implemented in R version 3.6.1^39^ using the mgcv package version 1.8–29^37,38^. The models were implemented using a binomial distribution and logit link function. The final presented plots are from the selected model fitted to all five folds. Variance Inflation Factors and concurvity values were calculated to ensure no multicollinearity was present between variables. Model validation was conducted by visual inspection of the residuals^40^.

4 $$\begin{aligned} BS= & {} \frac{1}{N} \sum _{t=1}^{N}(f_{t} - o_{t})^2 \end{aligned}$$

5 $$\begin{aligned} BS_{max}= & {} mean(p) \times (1-mean(p))^{2} + (1-mean(p)) \times mean(p)^{2} \end{aligned}$$

6 $$\begin{aligned} BS_{scale}= & {} \frac{1 - BS}{BS_{max}} \end{aligned}$$

Runners with missing data were removed from the analysis. The final dataset included 619,856 runners of which 659 were EHI incidents. Due to the large size of the dataset, in order to ensure the model was computationally tractable the non-EHI events in the training sets were randomly down-sampled such that the incidence of EHI was increased from 0.1 to 10%. This is similar to using a case-control approach under which the invariance of the model coefficients and odds ratios has been shown by mathematical proof^41^. However, it is not possible to make inferences about absolute probabilities based on the intercept parameter^42^. In this study, it was possible to correct for this because the true and sampled fractions of the EHI and non-EHI events is known. Consequently, the absolute predicted probability of EHI was estimated using a class adjustment as described by Matloff^43^. Data points were not independent. Horses were included in the dataset multiple times as they ran at different race meets, and horses running at the same race meet were running under similar conditions. Therefore, the random variables of horseID and a raceMeetID were included in the final model.

To enable post-hoc comparison of the different categories within year, going, previous incident and race off time the package emmeans was used to calculate the odds ratios (OR), confidence intervals and Tukey adjusted p-values to compare the estimated marginal means^44^. For continuous variables in a GAMM the odds ratios are not constant across all values of the variable. Therefore, an example odds ratio is calculated for a specific interval of the variable using the package oddsratio^45^. To calculate the marginal means, log(odds) of the predictors, other than that of interest, are set to their mean values. The log(odds) are then transformed into the odds of EHI occurring for a given risk factor (i.e. the probability that EHI occurs divided by the probability of EHI not occuring) and the odds ratio calculated. This is the ratio between the odds of EHI occuring for two levels of a risk factor. Plots of each smooth term and partial effects plots from the final model were generated using the R package mgcViz^39,46^. Modelled predicted probabilities were generated and plotted using the function ggpredict from ggeffects^47^ and ggplot^48^.

### Prediction of exertional heat illness

Management of EHI in a regulatory setting requires an understanding of risk based on probability as much as a correct binary classification. Therefore, the model was assessed using both the Brier Score and AUC metrics. The predicted probabilities were used to predict if horses would or would not present with EHI for each fold of the 5-fold cross validation. Test folds were not down-sampled and therefore, reflected the dataset wide incidence of 0.1%. As described above, the performance of the probability estimates was examined using the Brier Score (Eq. 4) and its scaled version (Eqs. 5, 6)^49^. In addition, the ability of the model to correctly classify runners as presenting either with or without EHI was examined using the Receiver Operating Characteristic (ROC) curve and area under the ROC (AUC) value. An appropriate decision threshold value for classification of EHI events was selected by finding the optimal balance between the true positive rate (TPR) and false positive rate (FPR). This was determined by calculating the G-mean ($G{\text -}mean = \sqrt{TPR \times (1-FPR)}$) for all possible thresholds between 0 and 1 with a increment of 0.01^50^. The chosen threshold was the one that maximised the G-mean value^50^. The results were visualised using an ROC curve and classification matrix generated with the ROCR^51^ and ggplot^48^ packages in R^39^.

## Results

### Risk factors for exertional heat illness

Exertional heat illness (EHI) was reported in 0.1% of runners between 1st July 2010 and 30th April 2018. This was higher for jump racing when compared to flat racing at 0.2% and 0.02% of runners respectively. The final selected model, based on backward model selection (Table 2), contained the variables race distance, WBGT, preceding 5-day temperature average, age, if there had been a previous EHI incident, the reported going, year and the race off time. Model validation plots and raw model coefficients are presented in the Supplementary Results. Despite the differences in incidence by race type, it was not included in the model as an explanatory variable during variable selection.

**Table 2:** Mean Brier Score and mean scaled Brier Score (%) for the assessment of candidate binomial GAMMs. Model selection was conducted by removing non-significant variables with backward selection. Models were assessed using 5-fold cross validation. Standard deviation is given in brackets. The removal of the variables sex and race type result in minor changes in the performance of the models as given by the Brier Score. Hence their inclusion in the model was not supported because they did not improve model performance. EHI refers to exertional heat illness.

Model	Brier Score	Scaled Brier Score	$\Delta$ Scaled Brier
M1: Full$^{\rm a}$	0.0148 (0.0007)	16.9446 (0.2706)	
M2: M1 - Sex$^{\rm b}$	0.0149 (0.0006)	17.0211 (0.2906)	0.0765
M3: M2 - Race Type$^{\rm c}$	0.0149 (0.0006)	17.0019 (0.2813)	-0.0192

$${}^{\rm a}$$ $$EHI \sim s(Age) + s(Distance) + s(WBGT) + s(5DayAverage) + Going + PreviousInc + Year + RaceType + RaceOff + Sex$$

$${}^{\rm b}$$ $$EHI \sim s(Age) + s(Distance) + s(WBGT) + s(5DayAverage) + Going + PreviousInc + Year + RaceType + RaceOff$$

$${}^{\rm c}$$ $$EHI \sim s(Age) + s(Distance) + s(WBGT) + s(5DayAverage) + Going + PreviousInc + Year + RaceOff$$.

**Figure 1:**
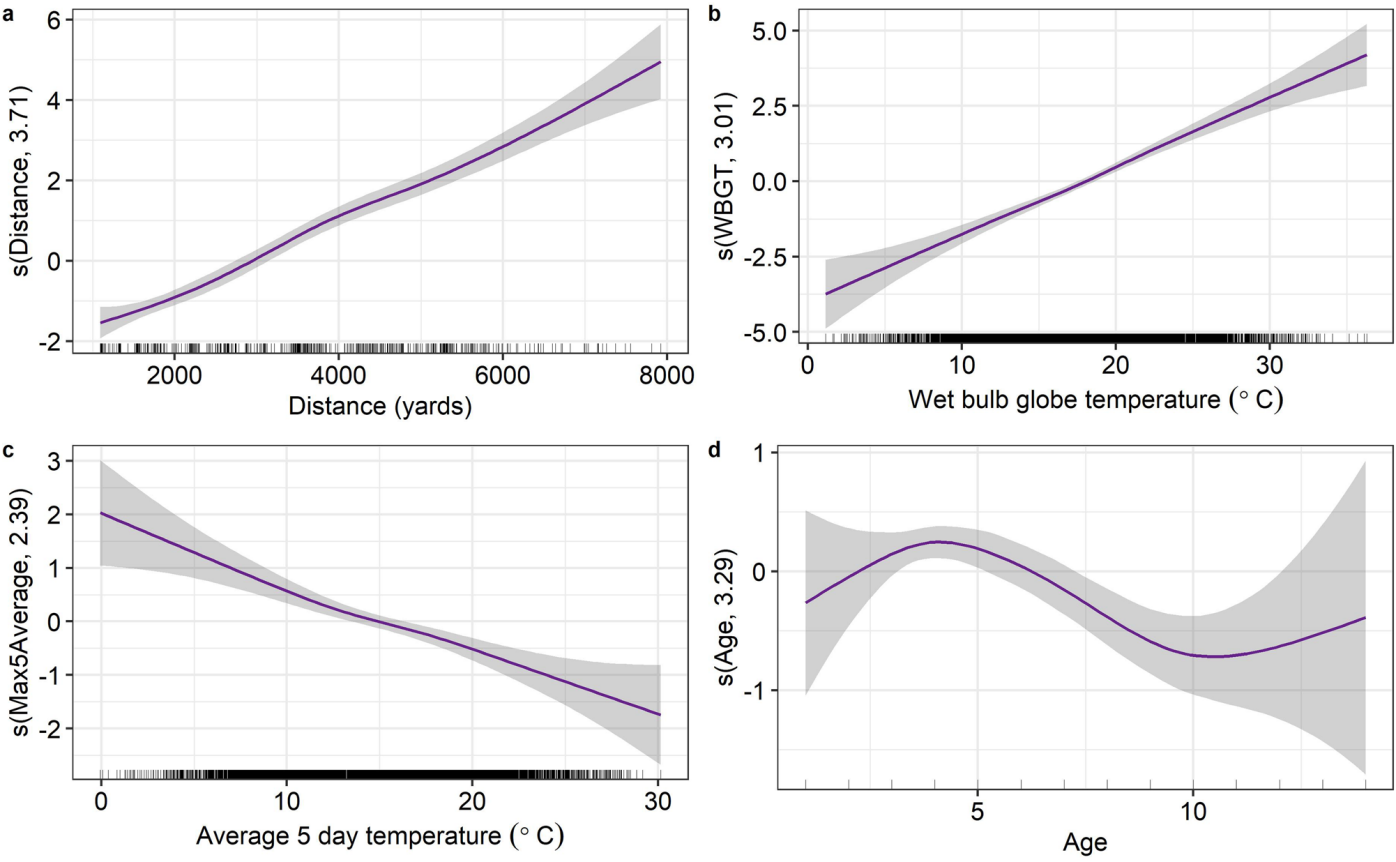
Estimated smooth terms (log(odds)) for generalised additive mixed model of the occurrence of exertional heat illness in Thoroughbred racehorses and model variables; race distance (**a**), wet bulb globe temperature (**b**), preceding 5-day temperature average (**c**) and age (**d**). The smooth is given by the purple line and 95% confidence intervals are displayed in grey shading. Rug plots show the distribution of raw data points.

An increase in the race distance was associated with an increase in the odds of EHI (edf = 3.71, $p < 0.0001$) (Fig. 1). For example, the odds of horses running a 2 mile race presenting with EHI were 5.66 (95% confidence interval (CI) 5.06–6.33) times the odds of horses running a 1 mile race. Similarly, an increase in the WBGT index was associated with an increase in the odds of EHI (edf = 3.01, $p < 0.0001$) (Fig. 1). For example, the odds of horses running at a WBGT value of 30 $^{\circ }$C presenting with EHI were 10.14 (95% CI 8.50–12.09) times the odds of horses running at a WBGT value of 20 $^{\circ }$C. In contrast, the preceding 5-day temperature average at the racecourse was associated with lower odds of EHI (edf = 2.39, $p < 0.0001$) (Fig. 1). For example, the odds of EHI for horses running in a race with a preceding 5-day temperature average of 25 $^{\circ }$C were 0.33 (95% CI 0.26–0.41) times the odds of horses running in races with a preceding 5-day temperature average of 15 $^{\circ }$C. The preceding 5-day temperature average at the racecourse did not differ from the horses training location for the majority of horses in a sample of 182,051 horses (Supplementary Results; Supplementary Fig. S2). Horses younger than 4 were associated with an increase in the odds of EHI and horses older than this were associated with a decrease in the odds of EHI (edf = 3.29, $p < 0.0001$) (Fig. 1). For example, the odds of EHI in 4 year old horses was 1.34 (95% CI 1.13–1.58) times the odds of EHI in 2 year old horses. The odds decrease for horses between 4 and 6 years old (OR = 0.82, 95% CI 0.80–0.84). The odds may increase in horses older than 10 but the low number of horses above this age in the dataset results in unacceptable uncertainty.

**Figure 2:**
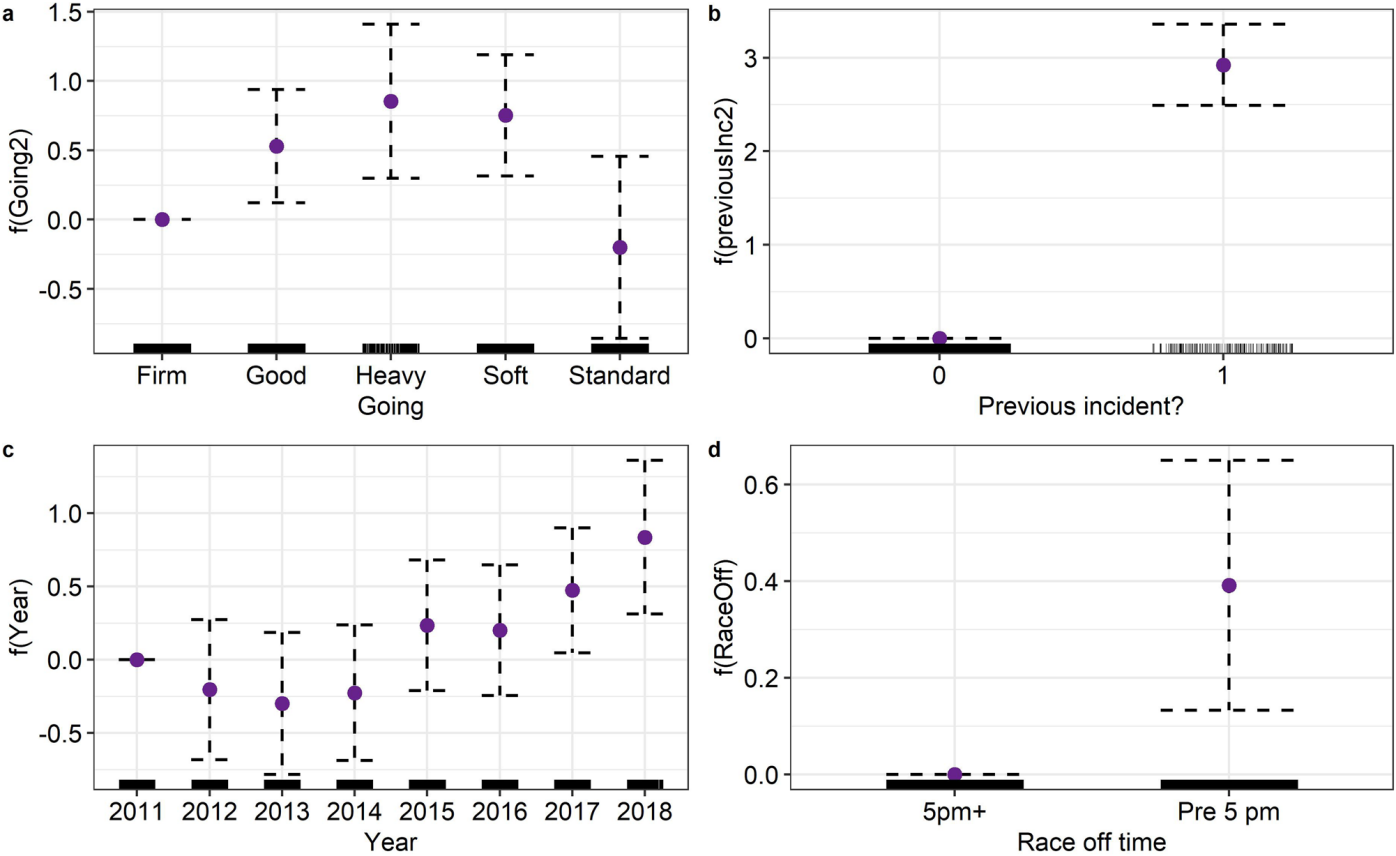
Partial effect plots for generalised additive mixed model for the occurrence of exertional heat illness (EHI) in Thoroughbred racehorses and model variables; going (**a**), if the horse had a previous EHI incident (**b**), year (**c**) and race off time (**d**). The value is given by the purple point and 95% confidence intervals are displayed by the dotted line. Rug plots show the distribution of raw data points.

Horses that had a previous EHI incident were more likely to have an EHI incident than horses that had not had a previous EHI incident (Fig. 2). The odds that horses with one or more previous EHI incidents would present with EHI were 18.59 (95%CI 12.00–28.70, $p < 0.0001$) times the odds of horses that had not had a previous EHI incident (Table 3). The odds of EHI in horses running on *heavy* or *soft* going was just over two times the odds of horses running on *firm* ground (Fig. 2; Table 3). The odds of horses running on *heavy* or *soft* turf surfaces presenting with EHI were again more than two times the odds of EHI in horses running on an all weather surface with *standard* going (Table 3). The odds of horses having an EHI incident was greater in the years of 2017 and 2018 when compared to the years of 2012, 2013 and 2014 (Table 3, Fig. 2). Furthermore, the odds in the year of 2018 were also significantly greater than 2011 (Table 3). Horses running in races that began before 5 pm had greater odds of presenting with EHI than horses running in races that started after 5 pm (Table 3).

**Table 3:** Post-hoc pairwise comparisons of categories within variables year, going, race off time and previous exertional heat illness (EHI) incident. The table presents the number of horse performances in each category of the down-sampled dataset and the number of those that resulted in an EHI incident in brackets, odds ratios (OR), t-ratios, *p* values using tukey adjustment for multiple comparisons and lower (CI-L) and upper (CI-U) 95% confidence interval. * indicates p values less than 0.05.

Variable	Comparison	Performances (EHI incidents)	OR	CI-L	CI-U	t-ratio	*p* value
Previous incident	Yes/no	177 (134) versus 7072 (525)	18.59	12.00	28.70	13.197	<.0001*
Race off time	Pre 5 p.m./after 5 p.m.	4766 (530) versus 2483 (129)	1.478	1.140	1.910	2.964	0.003*
Going	Firm/Good	1055 (41) versus 1935 (274)	0.589	0.334	1.041	− 2.538	0.083
	Firm/Heavy	1055 (41) versus 391 (60)	0.426	0.197	0.922	− 3.015	0.022*
	Firm/Soft	1055 (41) versus 2278 (269)	0.471	0.257	0.865	− 3.377	0.007*
	Firm/Standard	1055 (41) versus 1590 (15)	1.222	0.491	3.045	0.600	0.975
	Good/Heavy	1935 (274) versus 391 (60)	0.723	0.402	1.299	− 1.512	0.555
	Good/Soft	1935 (274) versus 2278 (269)	0.800	0.561	1.140	− 1.721	0.421
	Good/Standard	1935 (274) versus 1590 (15)	2.075	0.909	4.736	2.413	0.112
	Heavy/Soft	391 (60) versus 2278 (269)	1.107	0.652	1.878	0.522	0.985
	Heavy/Standard	391 (60) versus 1590 (15)	2.870	1.130	7.289	3.087	0.017*
	Soft/Standard	2278 (269) versus 1590 (15)	2.594	1.149	5.854	3.195	0.012*
Year	2011/2012	607 (43) versus 1019 (58)	1.228	0.586	2.575	0.842	0.991
	2011/2013	607 (43) versus 1020 (55)	1.349	0.638	2.855	1.211	0.929
	2011/2014	607 (43) versus 1051 (69)	1.254	0.614	2.563	0.961	0.980
	2011/2015	607 (43) versus 977 (99)	0.791	0.397	1.576	− 1.031	0.970
	2011/2016	607 (43) versus 1064 (98)	0.819	0.411	1.633	− 0.878	0.988
	2011/2017	607 (43) versus 1184 (158)	0.624	0.322	1.208	− 2.164	0.374
	2011/2018	607 (43) versus 327 (79)	0.434	0.193	0.976	− 3.124	0.038*
	2012/2013	1019 (58) versus 1020 (55)	1.098	0.558	2.163	0.420	1.000
	2012/2014	1019 (58) versus 1051 (69)	1.021	0.537	1.941	0.099	1.000
	2012/2015	1019 (58) versus 977 (99)	0.644	0.349	1.190	− 2.172	0.369
	2012/2016	1019 (58) versus 1064 (98)	0.667	0.361	1.231	− 2.006	0.478
	2012/2017	1019 (58) versus 1184 (158)	0.508	0.285	0.906	− 3.548	0.009*
	2012/2018	1019 (58) versus 327 (79)	0.353	0.170	0.733	− 4.326	0.0004*
	2013/2014	1020 (55) versus 1051 (69)	0.930	0.484	1.787	− 0.339	1.000
	2013/2015	1020 (55) versus 977 (99)	0.586	0.313	1.098	− 2.581	0.163
	2013/2016	1020 (55) versus 1064 (98)	0.607	0.326	1.131	− 2.433	0.225
	2013/2017	1020 (55) versus 1184 (158)	0.462	0.256	0.835	− 3.955	0.002*
	2013/2018	1020 (55) versus 327 (79)	0.322	0.153	0.677	− 4.620	0.0001*
	2014/2015	1051 (69) versus 977 (99)	0.631	0.353	1.128	− 2.404	0.240
	2014/2016	1051 (69) versus 1064 (98)	0.653	0.364	1.170	− 2.215	0.343
	2014/2017	1051 (69) versus 1184 (158)	0.497	0.288	0.858	− 3.887	0.003*
	2014/2018	1051 (69) versus 327 (79)	0.346	0.170	0.705	− 4.519	0.0002*
	2015/2016	977 (99) versus 1064 (98)	1.035	0.595	1.799	0.187	1.000
	2015/2017	977 (99) versus 1184 (158)	0.788	0.472	1.315	− 1.409	0.853
	2015/2018	977 (99) versus 327 (79)	0.548	0.277	1.086	− 2.667	0.133
	2016/2017	1064 (98) versus 1184 (158)	0.762	0.456	1.271	− 1.611	0.744
	2016/2018	1064 (98) versus 327 (79)	0.530	0.267	1.054	− 2.802	0.095
	2017/2018	1184 (158) versus 327 (79)	0.696	0.362	1.336	− 1.686	0.696

**Figure 3:**
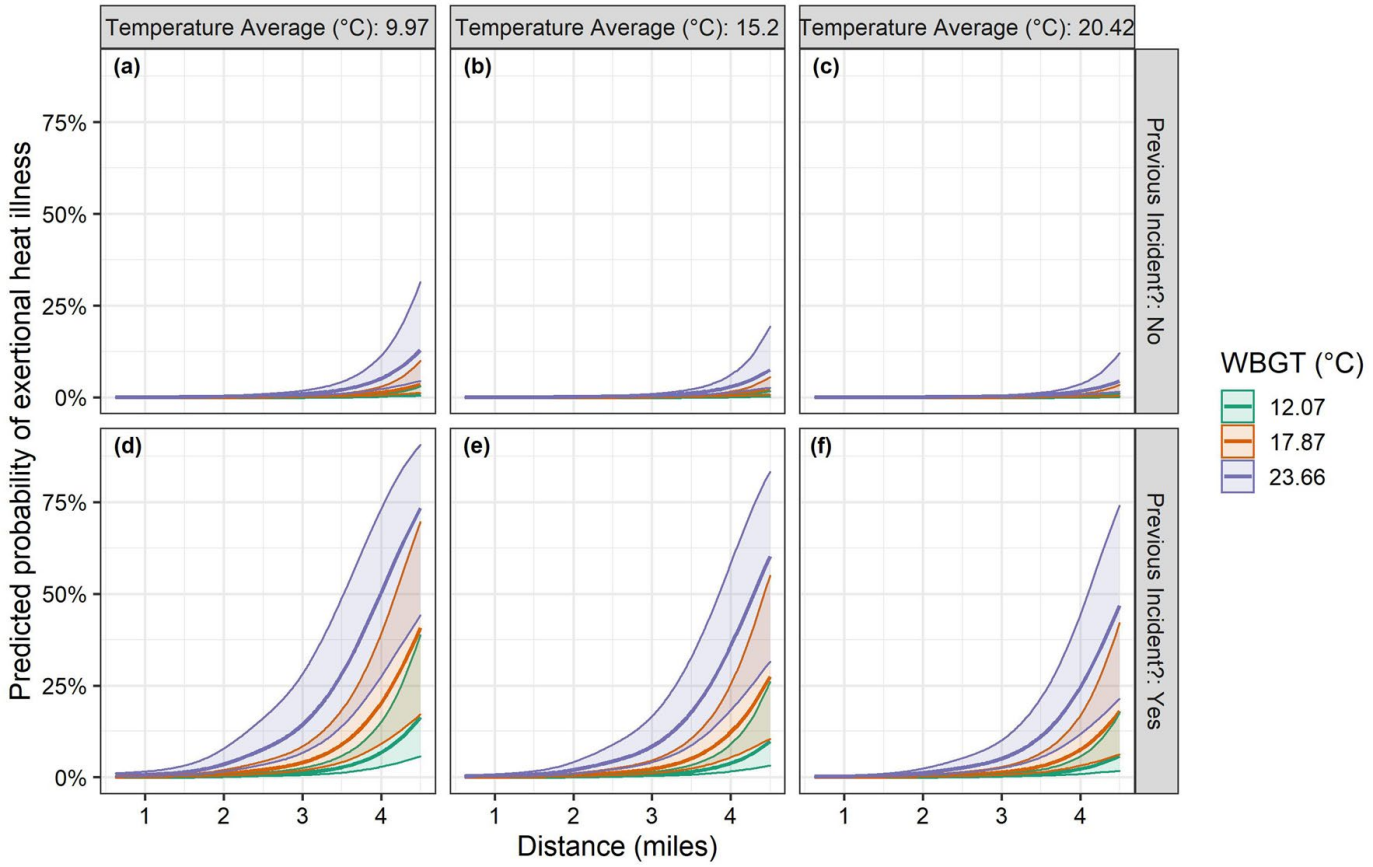
Modelled predicted probability of exertional heat illness by race distance at three values of wet bulb globe temperature (WBGT) ($^{\circ }$C) and preceding 5-day temperature averages ($^{\circ }$C) for horses that have not had a previous EHI incident (**a**–**c**) and those that have (**d**–**f**). Model variables not plotted are held at their mean value. Overall predicted probabilities are low under all conditions for horses that have not had a previous EHI incident. However, for horses that have had a previous EHI incident there is a greater risk of EHI during races across all scenarios.

The dataset wide incidence of EHI was low (0.1% of runners) and this was reflected in the absolute probability of EHI predicted by the model (Fig. 3). However, when horses have had a previous EHI incident, the probability of EHI in the horse’s current race exceeded 75% when running races over 4 miles long at a relatively low WBGT value of 23.71 $^{\circ }$C, especially when temperatures have been low in the five days leading up to the race (Fig. 3).

### Prediction of exertional heat illness

**Figure 4:**
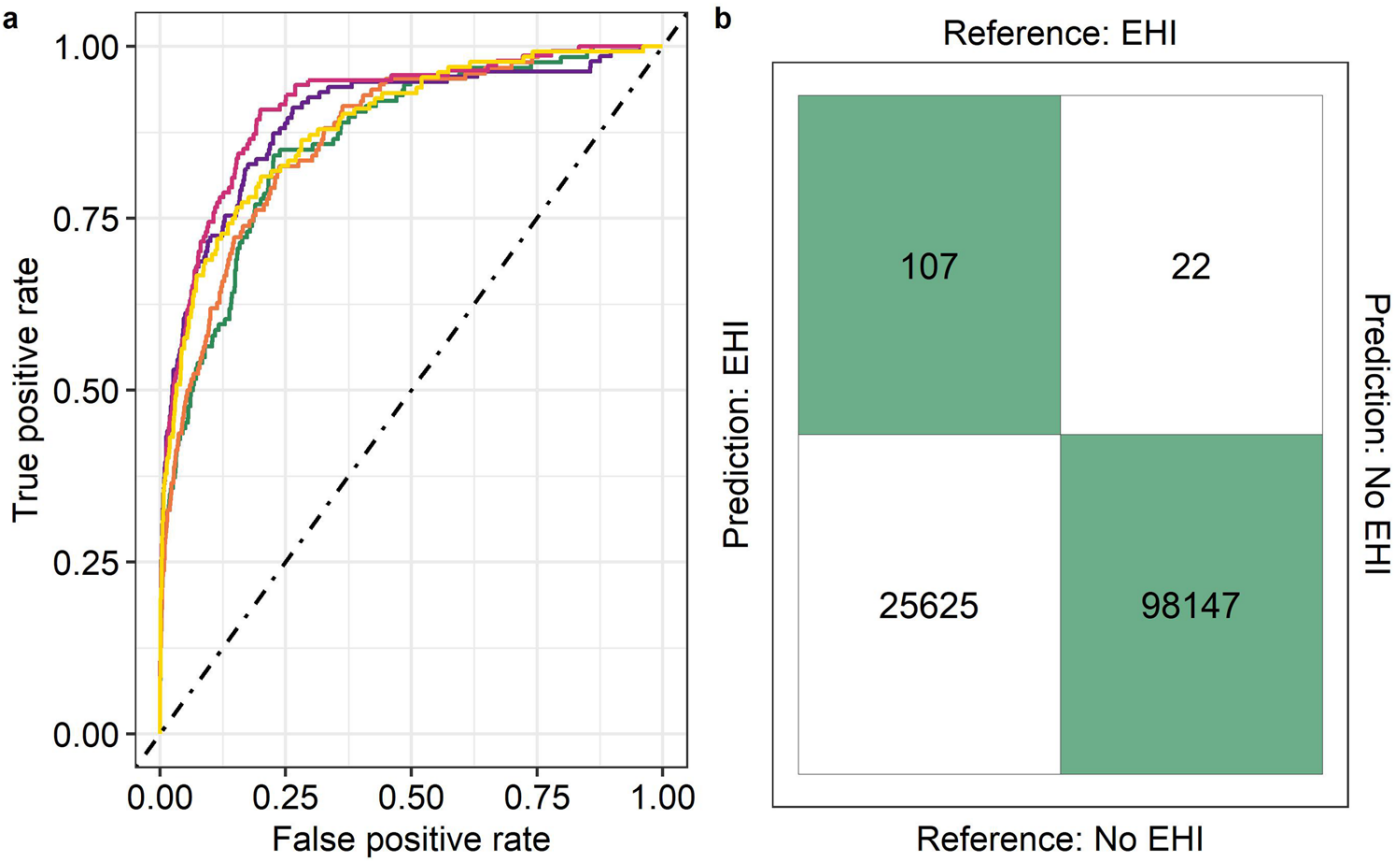
Receiver Operating Characteristic curve (**a**) and Classification Matrix (**b**) for prediction of exertional heat illness through 5-fold cross validation. Each line in Panel **a** represents a different fold. The confusion matrix represents the median values across all five test folds. The dashed line indicates the hypothetical model with no discrimination ability. The mean AUC value was 0.884 (SD = 0.02).

The mean scaled Brier Score for the final model was 17% (SD = 0.28). The decision threshold was optimised using the ROC curve and G-mean statistic. The mean decision threshold was 0.09 (SD = 0.02) and the mean AUC value was 0.884 (SD = 0.02) (Fig. 4). Under this scenario, 83.5% (SD = 4.40) of EHI incidents were identified (Fig. 4). However, the classification suffers from a large number of false positive predictions with only 0.44% (SD = 0.08) of EHI predictions actually resulting in an EHI event (Fig. 4). In each race several horses run under the same conditions but only a minority will present with EHI. Therefore, when considering identification of races in which 1 or more horses present with EHI the number of false positives can be marginally reduced with 0.8% of EHI predictions actually resulting in an EHI event.

## Discussion

The WBGT, previous 5-day temperature average, race distance, age, if the horse had a previous EHI incident, going, year and race off time, were associated with the probability of a horse presenting with EHI at British racecourses between July 2010 and April 2018. In agreement with previous reports, an increase in the WBGT was associated with greater odds of EHI^18,28^. The Fédération Equestre Internationale (FEI) utilise WBGT to characterise the environmental conditions under which equine events take place^29^. For FEI events, mitigation measures should be in place for WBGT values between 28 and 32 $^\circ$C. WBGT values above 32 $^{\circ }$C are thought to be hazardous and incompatible with safe competition^29^. In this study, 321 races were in these FEI harzardous or unsafe categories. The results show the odds of a horse presenting with EHI at a WBGT of 30 $^{\circ }$C were 10.14 times that of horses running at a WBGT of 20 $^{\circ }$C. The result suggests that officials should monitor WBGT at race meets to support effective decision making about the suitability of conditions for racing. Jurisdictions that already operate in climates with sustained high temperatures such as New South Wales, Australia, have used WBGT to define their policy on racing in extreme heat. In the policy, a WBGT of 26 $^{\circ }$C in the shade signals the need for initial mitigations and a WBGT value of 28 $^{\circ }$C in shade requires veterinary advice to continue^52^. However, the results of this study show that many EHI incidents occur at lower WBGT values. This highlights the commensurate importance of the other risk factors, such as a history of the condition, for the presentation of EHI. Furthermore, this result provides evidence that while high WBGT values require additional management, or the suspension of racing by officials, the provision of appropriate resources such as cool down areas with cold water for aggressive cooling, should not be limited to races with high WBGT.

The use of WBGT for decision making in equestrian sport is contentious^53^. WBGT was developed as an index of the environmental heat load placed on exercising military recruits^28,31^. It considers temperature and other environmental factors, such as wind speed and humidity, that can influence the ability of horses to lose heat to the environment^20^. WBGT was assessed for use in horses during 3-day eventing^28,31^, but it is unknown how WBGT relates to epidemiological data for racehorses^53^. Furthermore, evidence suggests wind speed and humidity may be better predictors for EHI compared to the composite WBGT value^20^. However, WBGT was utilised in this study because the environmental variables, temperature and humidity, were co-linear and it is known a simple addition of humidity and temperature is a poor predictor of EHI^20,28^. The results show a strong relationship between increasing WBGT and the presentation of EHI in racehorses. However, confidence intervals were wider at WBGT values above 25 $^{\circ }$C. This may be due to the lower number of races that take place under these more extreme conditions and the use of a WBGT estimation, rather than a measured value. WBGT was not collected at racecourses during this study. Therefore, a WBGT estimation was utilised which does not use a measure of wind speed. Despite this, even at the extremes of the upper and lower confidence intervals, there is still a positive relationship between increasing WBGT and the odds of a horse presenting with EHI, which is useful evidence for racecourse decision makers. In light of this finding, it is recommended racecourses collect temperature, humidity, wind speed and a composite measured WBGT to facilitate decision making, and to improve future iterations of the risk factor model.

Lower odds of EHI were associated with higher temperatures in the five days preceding a race. This suggests that some acclimatisation to warmer temperatures reduces the odds of EHI. The benefits of heat acclimatisation for reducing the detrimental physiological and performance manifestations of EHI is well reported in human athletes^54,55^. Through progressive repeated training in hot environments, adaptations such as decreased heart rate, decreased core temperature, and increased plasma volume can occur within three to five days of the onset of the acclimatisation process^54,56^. Although, the extent and speed of acclimatisation can be influenced by the type of training regime undertaken and many individual physiology parameters. In horses, physiological experiments support the finding of this study that acclimatisation before racing in the heat may be beneficial. Horses trained at temperatures over 30 $^{\circ }$C and a relative humidity (RH) over 80% for up to 21 days, increased their thermal tolerance, showing an increased ability to complete an exercise test, a decrease in the calculated total sweat ion losses due to a decreased sweating rate, a decrease sweat $Na^{+}$ concentration, lower resting body temperatures and lower heart rates^13,14,16,57^. These benefits were less pronounced when exercising in hot and humid conditions compared to hot and dry conditions^57^. Changes in plasma volume were more varied, with reports of both unchanged^14^ and increased plasma volume after 14 days^58^. Many of these studies undertake acclimation in artificial hot and humid conditions, which could be complex for trainers to replicate in practice. However, conditioning regimes under cool conditions can also enhance the ability of horses to tolerate hot conditions^16^. For untrained Thoroughbred racehorses, 10 days of training in cool conditions improved heat dissipation during an exercise test in hot conditions, demonstrating that fitness and conditioning can play a role in the risk of EHI^16^. However, data on training regimes were not available as part of this study. It would be useful to consider fitness as a risk factor for EHI in future models to potentially improve the predictive ability of the model. Information about horse fitness is difficult to collect given its central role in horse competitiveness. Possible proxies include the number of races the horse has undertaken in the weeks before a race. However, there is little known about how such proxies may relate to horse fitness given the different practices among trainers, age groups and race types^59,60^. Such a relationship would need to be verified before use as a proxy for fitness in risk factor models. However, the results highlight that when regulators and trainers are assessing the conditions under which horses are racing, and the associated risk of EHI, the weather conditions from the preceding five days can be informative in making decisions.

To assess weather in the five days leading up to a race, this study utilised average maximum temperatures at the racecourse in the five days before a race. This variable was used because five days of acclimatisation to environmental conditions has been shown to produce significantly lower rectal temperatures than horses on day 1 of acclimatisation^13^. It was also, and will continue to be, easily available to racecourse decision makers. This value may differ from the temperature at the horses location in the five days before a race. However, the differences are negligible for the majority of horses. This assumption was tested for a subset of horses whose pre-race location was known. Temperature between the racecourse and the horses location in the five days before a race differed within ± 2.7 $^{\circ }$C for 95% of horse and within ± 1 $^{\circ }$C for 61.6% of horses. This suggests that the preceding 5-day temperature at the racecourse is a good estimate of the pre-race conditions for most horses (Supplementary Fig. S2).

For exercising horses, exercise workload is a key determinant of internal heat production^1,2^. An increase in race distance and heavier going both increased the odds of horses presenting with EHI. These factors are both linked to the workload that horses are undertaking. Similarly, models of metabolic heat production at 3-day events suggested heat production was determined by exercise duration, which was predicted by the distance run and the running speed^11^. In the endurance horse, the odds of failing to pass vet gates during a competition was increased for horses competing over 120 km compared to those competing over 80 km^61^. Furthermore, horses competing at faster riding speeds (19.7 km/h vs. 15.6 km/h) also had greater odds of failing to pass the vet gates due to metabolic issues^61^. While metabolic issues are broader in scope than the condition of EHI, it does encompass heat-related conditions. In eventing, varying the course length has been used to reduce the exercise workload^29,62^. The results presented here support the use of similar options, such as reducing race distance as a method by which the risk of EHI for racehorses could be reduced, especially in high risk environmental conditions. Race speed would be more difficult to manipulate in order to reduce the risk of EHI. However, it could also be a useful additional variable in predicting EHI if it was to be included in future regulatory data holdings. It should be noted confidence intervals for model estimates of going are wide due to a low number of incidences for races run over firm and standard going. The magnitude of the possible impact of heavier going is uncertain and consequently it is not appropriate to give recommendations based on this result.

Race type and sex were not included in the final model. For race type, the difference in incidence was explained by other factors such as the age of the horse and the environmental conditions under which certain race types are predominantly run. Similarly, the results showed no significant difference in the probability of EHI by sex. Evidence does not support sex differences in incidence of EHI in human athletes when factors such as individual anthropometric characteristics are considered^55^. In contrast, for Thoroughbred horses running flat races in Japan, geldings and mares were at higher risk of EHI than uncastrated males^18^. This difference could be because the majority of uncastrated males as part of this dataset are younger horses and age was identified as a risk factor in this study. Horses had a higher risk of EHI as a 4 year old but beyond this the risk of EHI decreased with age. Older horses are thought to be at greater risk of EHI due to ageing related impairment to the cardiovascular system. Old horses have been shown to reach a core temperature of 40 $^\circ$C more quickly and have faster heart rates compared to young horses undertaking exercise^17^. The young horses also had a greater plasma volume^17^. However, the horses studied here had a very different profile than those used by McKeever et al.^17^. As highlighted in the results, uncertainty is too great for horses over 10 years of age to draw any conclusions from the results. This is due to the low number of horses that race above this age. In McKeever et al.^17^ the mean age of horses was 26.6 and 7.7 years for old and young horses respectively. Horses were also unfit standardbreds. In contrast, the racehorses in this study had a median age of 5 (1 - 14) and were all in training, and so were closer in age to the young cohort than the old cohort of McKeever et al.^17^. An increase in EHI up to 4 years of age may be explained by a lower level of fitness in the younger horses and an increasing workload as horses develop. In humans it is reported that age related thermoregulatory diminishments are less in fitter individuals^63,64^. The older horses could therefore, benefit from being more highly trained than the younger horses. Older horses could also be self selecting in that they are the horses that have the traits to succeed and therefore, still be racing at an older age which could contribute to their lower risk.

A proportion of horses are having multiple incidents throughout their careers. If a horse had a previous EHI incident the odds of presenting with EHI was 18.59 times the odds of horses that had not had a previous incident. This relationship was anecdotally reported by McCutcheon and Geor^2^ but is supported here by data. There was only 1 additional horse level variable (age) included in the model so it is difficult to determine the driver of repeated EHI incidents and establish if horses were always predisposed to EHI or if something about the initial EHI incident made horses more susceptible to EHI in the future. In human athletes, there is recognition that a wide range of individual factors may heighten the risks to individuals. These include body fat, surface to mass ratio, $VO_{2max}$ and sweat rate^55^. Furthermore, there is evidence of a relationship between genetic factors and exercise heat tolerance. The angiotensin I-converting enzyme (ACE) I+ polymorphism has been associated with increased heat tolerance in humans^65^. For racehorses, there are a number of potential risk factors that relate to individual management that are not measured but that could be mediating this response such as behaviour, distance travelled to racecourse, education of staff, the practice of withholding of water and fitness regime. Hydration is a key risk factor for EHI in humans^55^. Dehydration reduces the ability of horses to dissipate heat because it reduces conductance of heat from the core to the periphery^2^. In some jurisdictions, withholding water before a race or administration of diuretic furosemide is permitted to prevent exercise induced pulmonary haemorrhage^66^. In South Africa, horses administered furosemide were 5.5 times more likely to experience EHI than horses that were not administered the drug^67^. Particularly, it is clear from the results that identifying horses that have had a previous EHI event to racecourse veterinary officers would provide the opportunity to prevent immediate EHI incidents, target early treatment or engage with trainers and owners regarding future management of susceptible horses. The risk was higher for horses with previous incidents at lower WBGT values and shorter race distances, suggesting intervention for these horses is required before intervention for the racing cohort as a whole.

The results show an increase in EHI incidents in 2017 and 2018 compared to 2012, 2013 and 2014, and 2018 compared to 2011. Japan has also reported an increase in EHI incidents^24^. Prevalence between 1999 and 2018 was 0.04% but in 2017 and 2018 the prevalence exceeded 0.07%^24^. This rise was attributed to increasing awareness of EHI as a potentially lethal condition, a 1.19 $^{\circ }$C average temperature increase in Japan over the last 100 years and an increase in the number of days with a maximum temperature greater than 35 $^{\circ }$C^24^. Similarly, in the UK 2009 - 2018 was 0.9 $^{\circ }$C warmer than 1961–1990^68^. For England, 2018 was the equal warmest year on record since 1884, with the summer of 2018 being the sunniest 3 month period on record at that time^68^. While there was only partial data for 2018 in this study, a general increase in temperatures and EHI suggests more weather heat extremes may result in a greater number of EHI events, especially without mitigation in place. The increase could also be attributed in part to improving awareness of EHI and racecourse veterinary officers recording a wider variety of racehorse veterinary incidents. Despite this, the link between WBGT and EHI further supports the association between a greater number of heat wave events and increasing EHI. The chance of experiencing a summer as hot as 2018 is projected to be as much as 50% by 2050^69^. Furthermore, hot spells, defined as 2 or more consecutive days when daytime temperatures exceed 30 $^{\circ }$C, are projected to increase from 0.25 occurrences per year in the present day to 4.3 occurrences by 2070^69^. Globally, there has been 0.85 (0.65–1.06) $^{\circ }$C warming between 1880 and 2012 and an increase in warm climate extremes is predicted under future climate projections^70^. As such, racing jurisdictions will need to place an emphasis on the management of climatic extremes to safeguard horse welfare. The sharing of global best practice between differing climatic zones could facilitate effective management of EHI and assist in building climate resilience.

The model correctly predicted a high proportion (83.5%) of EHI events with a mean AUC value of 0.884 (SD = 0.02). However, the model would benefit from a smaller number of false positives and reduced uncertainty around model estimates^35^. While many horses raced under the same high risk conditions, the model could not determine which horses would be susceptible to those conditions. This was due, in part, to the inclusion of only two horse level explanatory variables in the model and the low incidence of EHI within the population. These are the key limitations of this study. The two horse level variables included in the final model were age and if the horse had a previous EHI incident. However, as discussed above, there are a large number of candidate variables that could further explain individual risk of EHI. These are not currently measured, or would be difficult to measure on the scale required for inclusion in a regulatory database. The results indicate the limited usefulness of the model for predicting EHI given the high number of false positives. The low incidence of EHI in the dataset was addressed by down-sampling the dataset to increase the incidence from 0.1 to 10%. The dataset contained all incidences of EHI for the study time frame. Therefore, collecting more data was not possible. The extent to which the data was sampled was a balance between retaining the information provided by the full dataset, convergence of the model and ensuring a balance of data across the categories of the explanatory variables. The low incidence of EHI still resulted in wide confidence intervals around model estimates for going, horses over 10 years of age and horses that have has a previous EHI incident and the results should be viewed cautiously in this context.

The racecourse was not included as an explanatory variable because it was co-linear with variables that explained the workload of horses more exactly such as race type and race distance. However, there are certain racecourse characteristics that could be collected in future that may influence the probability of a horse presenting with EHI. These primarily relate to facilities such as the availability of shade, the availability of water for cool down areas, the distance between the track and the cool down facilities and the ventilation of stables, all of which can vary considerably between courses^3,71^. Climate change projections suggest that all courses will require a minimum set a facilities to ensure horse welfare can be maintained. While the focus of this study has been exertional heat illnesses, environmental heat inputs alone can also cause heat related illnesses without exercise^2^. Hence, stabling facilities at high WBGT values could also pose a risk to equine welfare and contribute to the thermoregulatory load of horses prior to exercise. Pre-cooling event horses exercising at moderate WBGT values increased the time taken for horses to reach a critical temperature threshold^72^. Therefore, the conditions under which horses have been prior to exercise will have an impact on their heat balance, and providing appropriate resources and facilities offer further opportunities to mitigate the risk of EHI.

Environmental parameters were associated with greater odds of EHI. The input data were based on historic records of temperature and relative humidity. While gridded climatic data allowed the calculation of temperature at the location of the racecourse^32^, this approach is still associated with uncertainty^18^. In order to make decisions on the race day, instruments at the track and in stables would provide more accurate information on the conditions experienced by horses. For planning purposes, weather forecasts are important. However, weather forecasts are also associated with a level of uncertainty. If forecasts were used to predict EHI, this uncertainty should be quantified to ensure appropriate decisions can be made^73^. Such decisions may be related to the scheduling of races in the day. Races that started after 5 p.m. were associated with lower odds of EHI. This could be because the evening is likely to be the cooler part of the day, although fewer races were also scheduled at this time resulting in wide confidence intervals. Measurements of WBGT in the USA showed peak WBGT values between 12 p.m. and 4 p.m.^74^. In human sport and for some equestrian disciplines, the cancellation and rescheduling of events based on environmental parameters is already a recommended practice to safeguard athletes^29,75^. The results provide evidence similar practices could used to reduce the risk of EHI for racehorses. The WBGT values input in the model were derived from daily maximum temperature values. Measurements of WBGT on a finer scale could help provide more detailed evidence to improve model estimates and provide evidence on the most appropriate time of day for races to start in order to minimise the risk of EHI.

## Conclusion

The environmental conditions (WBGT and five day temperature average), workload (going and race distance), timing of the race (race off time and year) and individual susceptibility (age and previous EHI incident), were risk factors for horses presenting to racecourse veterinary officers with exertional heat illness. Increasing WBGT, heavy going, a low temperature in the preceding five days, increasing race distance, races run before 5 pm and races run in 2017 and 2018 were associated with greater odds of EHI in horses at British racecourses. In horses up to 4 years of age there was an increased risk of EHI but this declined in older horses. It was possible to predict over 80% of EHI incidents but the model produced a high number of false positives demonstrating a weak ability to pick out susceptible horses from those running under the same conditions. There was high uncertainty in model estimates, particularly for the variables going, race off time and age. Nevertheless, the results provide evidence for a number of recommendations to the racing industry. Particularly, it is evident that horses that have had previous EHI incidents should be identified to racecourse veterinary officers to allow for greater monitoring and early intervention. The strong association of WBGT and environmental conditions with EHI in racehorses suggests that meteorological measurements at the racecourse would be valuable for decision making before and during race meetings. This could include changes to racing schedules and race distance. Furthermore, the addition of data streams relating to individual horse fitness and characteristics such as weight could be considered to improve predictive capability. Cooling facilities are vital for the treatment of EHI, and while this study did not focus specifically on the provision of those facilities, it does highlight that the risk for some horses is high across a range of environmental scenarios. Therefore, facilities should be widely available not just during the most extreme conditions. As incidence increased in 2017 and 2018 in line with weather extremes, such policies would be in line with the the need for racing jurisdictions to mitigate the potential welfare consequences of a changing climate and develop the climate resilience of the sport. The results of this study are vital for the creation of evidence-based policy which will protect the welfare of racehorses under current and future climate scenarios.

## Supplementary Information

Supplementary Information.

## Data Availability

The datasets analysed during this study are not publicly available. However, data can be requested by contacting the British Horseracing Authority at dataprotection@britishhorseracing.com.
