# Supplementary material for: Risk factors for, and prediction of, exertional heat illness in Thoroughbred racehorses at British racecourses
Source: Sci Rep. 2023 Mar 14;13:3063. doi: 10.1038/s41598-023-27892-x (PMC10015008; doi:10.1038/s41598-023-27892-x)
Supplement: Supplementary file 1 — Supplementary Information. [file 41598_2023_27892_MOESM1_ESM.pdf]

Supplementary Information

Risk factors for, and prediction of, exertional heat illness in Thoroughbred racehorses at British racecourses

Leah E Trigg, Sally Lyons and Siobhan Mullan

Supplementary Methods

Classification of going

There were 10 going categories present in the dataset (Supplementary Tab. S1). There were a limited number of data points in some categories, which prevented convergence of the model. Therefore, the categories were aggregated to give 5 categories; firm, good, heavy, soft and standard. Supplementary Tab. S1 shows how the original data categories were allocated to the categories used in the model. There were only three race meets run over fast ground so these were removed from the dataset.

Supplementary Table S1: Amalgamation of going descriptors for input into models of exertional heat illness. The approach facilitated the convergence of the models.

Data Categories	Model Categories
Firm	Firm
Good to Firm	Firm
Hard	Firm
Good	Good
Heavy	Heavy
Good to Soft	Soft
Soft	Soft
Standard	Standard
Standard to Fast	Standard
Standard to Slow	Standard

Interaction variables

A possible interaction between wet bulb globe temperature on race day and the average temperature in the five days before a race was included in the initial model using a tensor product smooth. This was computationally intensive to include and was not a significant variable (edf=1.00, F=0.015, p=0.90) in the model. Therefore, it was removed from the model and the interaction not investigated further.

Supplementary Results

Model validation

A generalised additive mixed model (GAMM) was indicated because the logit was not clearly linearly related to each of the predictors (Supplementary Fig. S1). This was particularly evident for the relationship between the logit and age (Supplementary Fig. S1).

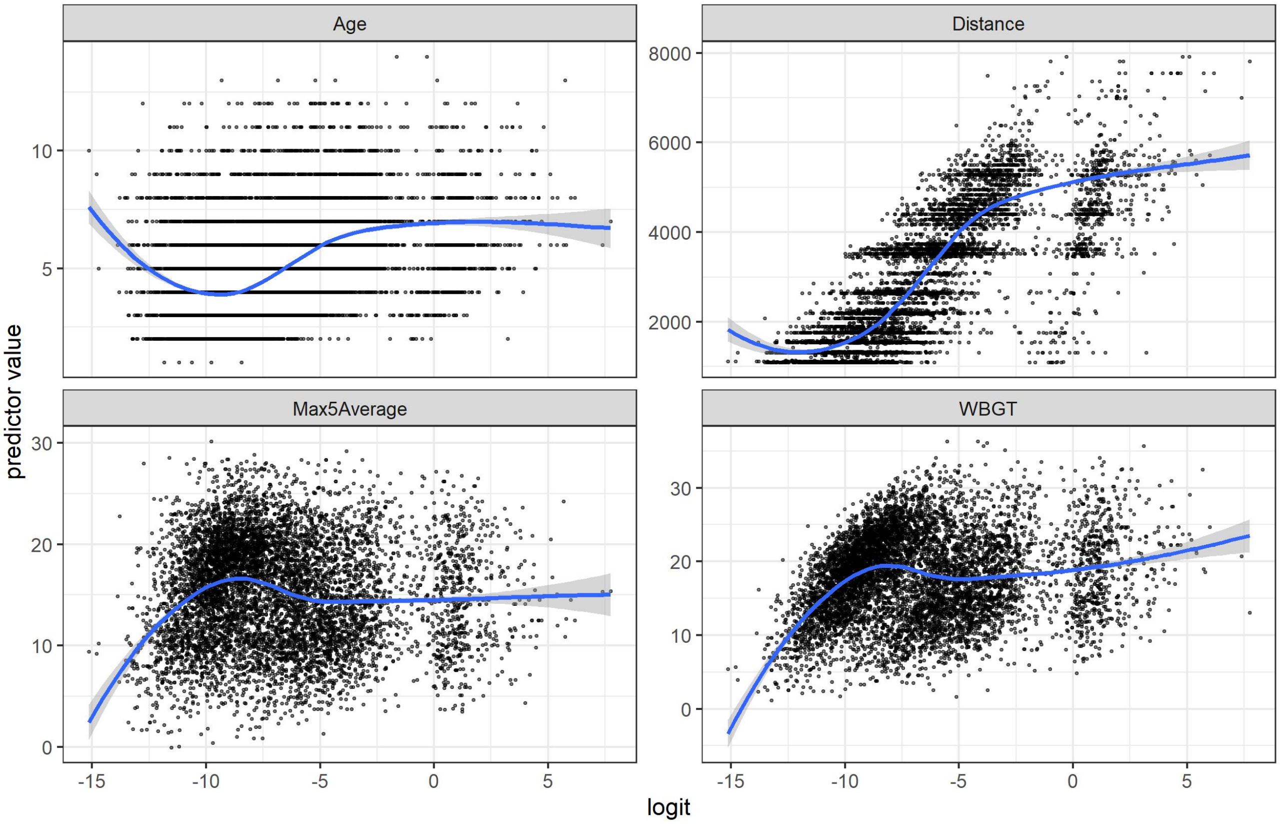

Supplementary Figure S1: Continuous variables plotted against the predicted logit value from a binomial generalised linear mixed model. The results show non-linearity between the logit and predictors. Age particularly cannot be considered linear and hence a generalised additive mixed model was considered more appropriate.

Variance Inflation Factors were used to remove variables with high levels of collinearity (Supplementary Tab. S2). The inclusion of wet bulb globe temperature, a composite of humidity and temperature, reduced collinearity between environmental variables. The variable month was removed because it was collinear with temperature and the type of racing that was being undertaken.

Supplementary Table S2: Variance inflation factors for variables included in the final selected model of exertional heat illness

Variable	VIF	Increased SE
Going	2.05	1.43
Previous Incident	1.04	1.02
Year	1.21	1.10
Race Off Time	1.16	1.08

Supplementary Table S3: Summary results of the final selected model of exertional heat illness in racehorses for parametric coefficients (estimates, standard error, t values and p values).

	Estimate	Std. Error	t value	Pr(>|t|)
(Intercept)	-4.256	0.277	-15.367	<0.0001
Going2Good	0.529	0.209	2.538	0.011
Going2Heavy	0.854	0.283	3.015	0.003
Going2Soft	0.752	0.223	3.377	<0.0001
Going2Standard	-0.200	0.335	-0.600	0.549
previousInc21	2.923	0.221	13.197	<0.0001
Year2012	-0.206	0.244	-0.842	0.400
Year2013	-0.300	0.247	-1.211	0.226
Year2014	-0.227	0.236	-0.961	0.337
Year2015	0.234	0.227	1.031	0.303
Year2016	0.200	0.228	0.878	0.380
Year2017	0.472	0.218	2.164	0.030
Year2018	0.835	0.267	3.124	0.002
RaceOffPre5pm	0.391	0.132	2.964	0.003

Supplementary Table S4: Approximate significance of the smooth terms included in final selected model of exertional heat illness.

Variable	edf	Ref.df	F	p-value
s(Age)	3.286	9	3.205	<0.0001
s(Distance)	3.706	9	32.736	<0.0001
s(WBGT)	3.008	9	20.277	<0.0001
s(Max5Average)	2.388	9	4.165	<0.0001

Previous 5-day temperature average

To assess the influence of acclimatisation on the probability of exertional heat illness, the risk factor model included the average maximum daily temperature at the racecourse in the 5-days before a race as an explanatory variable. However, the conditions at the racecourse may have been different to the conditions where the horse was located in the 5 days before the race. To assess the magnitude of this difference, the temperature difference between the location at the racecourse and at the horses location was calculated for a subset of 182,051 performances where the location of the horse in training was known. The mean difference in the average maximum temperature in the 5 days before a race performance at the training location and racecourse was -0.3 (SD=1.2) °C (Supplementary Fig. S2). The majority of performances (61.6%) were with ± 1 °C and 95% were within ± 2.7 °C.

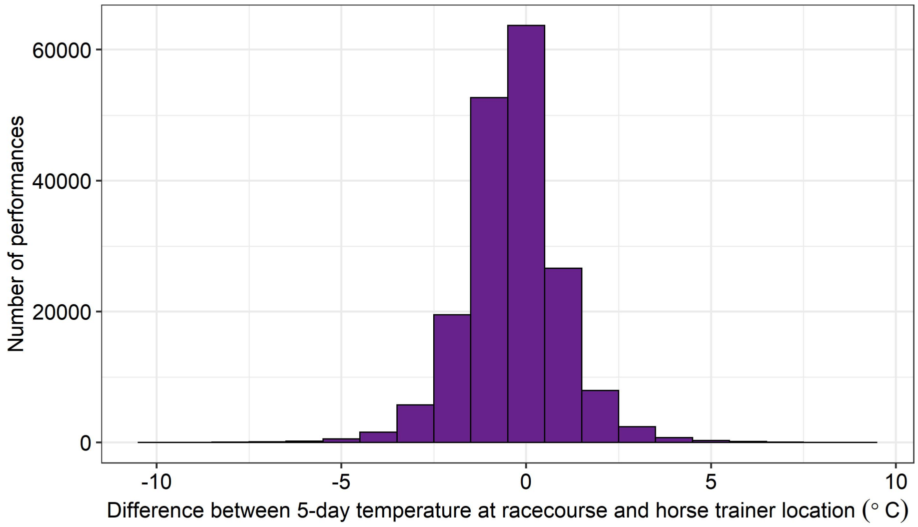

Supplementary Figure S2: Histogram of the differences in average 5-day maximum temperatures between horse training location and racecourse for a subset of 182,051 race performances where the training location of the horse was known.

The temperature at the racecourse was considered preferable as an explanatory variable because it will be easily available to racecourse decision makers. There may also be uncertainty in where the horses are kept compared to the trainers registered location.
